# High-throughput and quantitative genome-wide messenger RNA sequencing for molecular phenotyping

**DOI:** 10.1186/s12864-015-1788-6

**Published:** 2015-08-05

**Authors:** John E. Collins, Neha Wali, Ian M. Sealy, James A. Morris, Richard J. White, Steven R. Leonard, David K. Jackson, Matthew C. Jones, Nathalie C. Smerdon, Jorge Zamora, Christopher M. Dooley, Samantha N. Carruthers, Jeffrey C. Barrett, Derek L. Stemple, Elisabeth M. Busch-Nentwich

**Affiliations:** Wellcome Trust Sanger Institute, Wellcome Trust Genome Campus, Hinxton, Cambridgeshire CB10 1SA UK

**Keywords:** mRNA transcript profiling, RNA-seq, Molecular phenotype

## Abstract

**Background:**

We present a genome-wide messenger RNA (mRNA) sequencing technique that converts small amounts of RNA from many samples into molecular phenotypes. It encompasses all steps from sample preparation to sequence analysis and is applicable to baseline profiling or perturbation measurements.

**Results:**

Multiplex sequencing of transcript 3′ ends identifies differential transcript abundance independent of gene annotation. We show that increasing biological replicate number while maintaining the total amount of sequencing identifies more differentially abundant transcripts.

**Conclusions:**

This method can be implemented on polyadenylated RNA from any organism with an annotated reference genome and in any laboratory with access to Illumina sequencing.

**Electronic supplementary material:**

The online version of this article (doi:10.1186/s12864-015-1788-6) contains supplementary material, which is available to authorized users.

## Background

Analysis of the expression of mRNA produces a molecular readout of the biological activity in a tissue or entire organism sample, reflecting which parts of the genome are being transcribed and how much of each transcript is available for translation into protein or to perform a regulatory role. Comparing mRNA expression across different conditions, such as developmental stages or after gene perturbation, helps to unravel the complexities of biological systems. In recent years high-throughput short tag RNA sequencing technology (RNA-seq) has provided a convenient tool for delving deeper into mRNA expression [1] using the whole or part of each transcript [2–6]. More specifically, sequence reads are converted into count data with the aim of quantifying transcriptional differences between biological samples using polyA pull down at the 3′ end of transcripts [7–12] and cap analysis gene expression (CAGE) at the 5′ end of transcripts [13, 14].

Many RNA-seq methods are excellent tools for in-depth mRNA expression analysis of small numbers of samples and provide information on the entire RNA molecule, alternative splicing and the quantity of transcript. However, they require an involved library preparation and often complex sequence analysis [15] and are not amenable to large-scale application with a fast turn-around. We present a purely quantitative digital gene expression sample processing and analysis package called differential expression transcript counting technique (DeTCT) that begins with tissue samples and produces a text table or HTML table, comprising genomic coordinates representing the 3′ ends of genes, raw and normalised counts, and a fold change in transcript abundance between two conditions with an associated p-value. Our simplified library preparation and analysis protocol incorporates a sample indexing system and allows processing and sequencing of large numbers of samples and replicates. The genomic coordinates can be compared to existing gene annotation, but they also identify unannotated genomic regions showing an alteration in polyA+ transcript number. To assess the utility of the pipeline we used zebrafish mutants carrying loss of function alleles from the Zebrafish Mutation Project (ZMP) [16] and compared morphologically abnormal embryos with normal sibling embryos.

## Results and discussion

### Library preparation and sequence processing

**Fig. 1:**
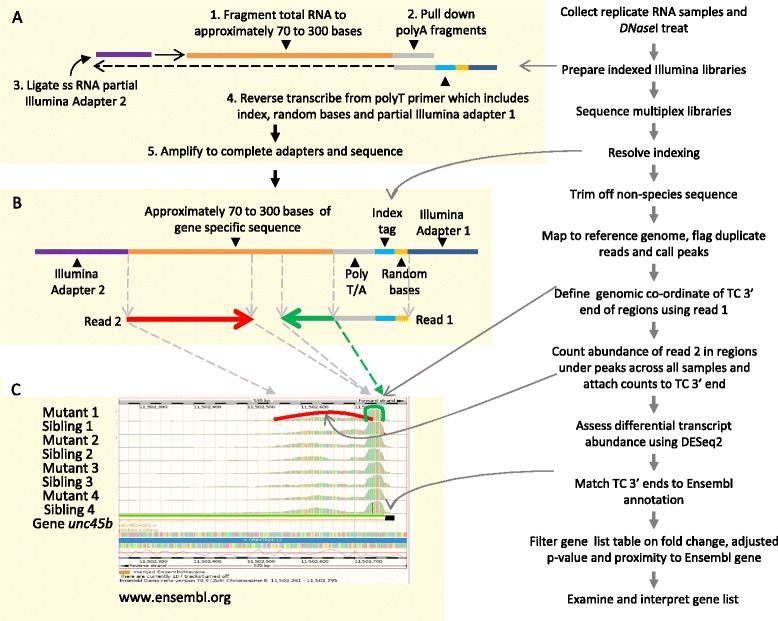
DeTCT pipeline workflow. Between nine and 11 pairs of mutant and normal zebrafish embryos were collected from one clutch and RNA extracted. **a** Following DNaseI treatment and chemical fragmentation, molecules representing the 3′ end of transcripts were enriched by pulldown using an anchored biotinylated oligo dT primer attached to streptavidin magnetic beads (orange line). An RNA oligo matching part of the Illumina read 2 adapter (purple line) was ligated onto the 5′ end, the RNA eluted and annealed to an oligo comprising partial read 1 Illumina adapter (dark blue line) followed by 12 random bases (beige line), then an eight base indexing sequence specific to each sample (light blue line) and finally a 14 base anchored polyT sequence (grey line). After reverse transcription the Illumina adapter sequences were completed during library amplification. Libraries were quantified, pooled in equimolar amounts and sequenced by Illumina HiSeq 2500. **b** After decoding the indexing sequence, the trimmed zebrafish sequences (read 1 in green and read 2 in red) were mapped to the reference genome and duplicate reads were flagged. **c** The coordinate representing the transcript counting 3′ end (TC 3′ end) was predicted using the base immediately 3′ of the polyT sequence in read 1 (green dashed arrow and green curved line). After calling peaks using all mapped read 2s the resulting counts were associated with their respective sample (red curved line). The count data were used to identify differential transcript abundance between conditions using DESeq2 [28] and reported as a fold change with an adjusted p-value. The TC 3′ ends were matched to the closest Ensembl transcript 3′ ends on the same strand (black line). Gene list tables were produced and ordered by the lowest adjusted p-value. These gene lists were filtered for genes showing differential transcript abundance using the adjusted p-value and the proximity of the TC 3′ end and Ensembl gene end (typically adjusted p-value <= 0.05 and within -100 and +5000 bases)

**Fig. 2:**
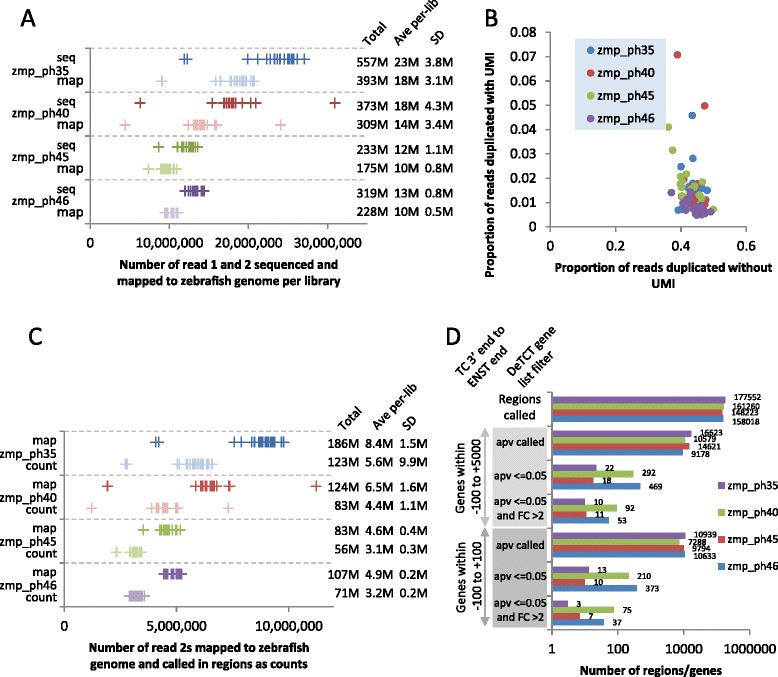
Library features. Four sets of libraries were prepared from zebrafish embryo lines carrying a disrupted gene. Morphologically abnormal and normal sibling embryos were collected from in-crossed lines and total RNA extracted. For each mutant line 18 to 22 samples of indexed libraries were made and sequenced by Illumina HiSeq 2500. **a** The number of reads and the number mapping to the Zv9 reference genome per library are shown. The total reads, the mean per library and the standard deviation are shown on the right. **b** For each library (dots) the proportion of reads identified as duplicates using outer mapping coordinates alone are shown on the x-axis and after accounting for the unique molecule identifier (UMI) on the y-axis. **c** The reads were passed through the DeTCT analysis pipeline. The number of read 2s mapped and the number of counts called under peaks as discrete regions are shown per library sample. The total reads, the mean per library and the standard deviation are shown on the right. **d** Using the gene list output from the DeTCT pipeline the chart shows the number of discrete regions identified per collection of libraries, the number of genes with an adjusted p-value (apv) from DESeq2 (i.e. those not removed due to low mean counts), the number of genes with an adjusted p-value <= 0.05 and the number of genes with an adjusted p-value <= 0.05 plus a fold change (FC) > 2. These analyses were performed with a stringent (-100 to +100) or relaxed (-100 to +5000) proximity filter between the Ensembl transcript and TC 3′ end

We selected four mutant zebrafish lines from the Zebrafish Mutation Project (ZMP) to test the differential expression transcript counting technique (DeTCT) pipeline. We collected morphologically normal and abnormal sibling single embryos in replicates from the same clutch obtained from timed single pair matings to synchronize the developmental stage. Total RNA was extracted from single zebrafish embryos with sufficient residual DNA to confirm the genotype of each embryo by KASP genotyping [17]. Libraries were prepared from 300 ng of total RNA. Several features make our libraries different to standard RNA-seq methods [15]. We have simplified the library preparation by reducing the number of steps, but have added several useful modifications (Fig. 1 and Additional file 1). The DNaseI digestion has been combined with the RNA fragmentation step and is followed by the first anchored polyA pulldown enrichment. While the RNA molecules are immobilised on magnetic beads RNA to RNA ligation introduces part of Illumina adapter sequence 2. After elution we perform a second round of 3′ end enrichment with an anchored oligo dT reverse transcription primer. This primer also incorporates a sample-specific in-read index sequence, a unique molecular identifier (UMI) sequence and part of Illumina adapter 1. After reverse transcription through the captured RNA molecules and the partial Illumina adapter 2 sequence, Illumina adapters are completed during a final library amplification step. The replicate libraries for each allele were pooled and sequenced on one lane equivalent (zmp_ph40, 45 and 46) or two lane equivalents (zmp_ph35) by Illumina HiSeq 2500. Figure 2a shows a sequence depth of between 373 and 233 million read pairs per lane equivalent, with 85 % and 76 % of the sequence mapping to the Zv9 zebrafish reference genome, respectively. Figure 2a also shows a relatively even quantity of sequence per library with the occasional outlier. During library preparation the sample amplification can result in duplicate reads for the same original transcript which is particularly relevant if an unknown amount of RNA is accidentally lost before amplification. The duplicate rate in a library reflects library complexity and is therefore an important quality indicator for each library. Duplicate reads caused by amplification can be identified by incorporating random sequences as unique molecular identifiers (UMI) into the primary sample [18–25]. We use a modified version of Picard MarkDuplicates [26, 27] and flag reads as potential duplicates if they share outer coordinates with other mapped read pairs and have the same UMI. Figure 2b shows that accounting for the UMIs reduced the median duplicate rate from 43.7 % to 1.2 % with a few libraries showing a higher duplicate rate of up to 7 %. If the duplicate rate goes above 20 % then we examine laboratory procedures for technical issues such as RNase contamination in reagents. This method remains an estimate of library duplication due to the possibility of two independent molecules carrying the same UMI and UMI sequence alteration during subsequent amplification. Read 1 is used to predict a single genomic position defining the transcript counting 3′ end (TC 3′ end). Between 66 % and 68 % of the read 2s map to the reference genome and where they accumulate peaks are called and reads are quantified as counts (Fig. 2c). Read pair information attaches these count data to the TC 3′ end and the in-read index sequence identifies their sample origin. DESeq2 [28] is used to estimate differential transcript abundance between conditions, independent of gene annotation. While the strand-specific TC 3′ ends can be linked to any gene annotation, here we use the Ensembl gene build [29]. Fig. 2d shows the total number of regions called as peaks (mean 161,263), the subset associated with a gene where DESeq2 has estimated an adjusted p-value (i.e. where the total counts are sufficient to potentially distinguish between conditions), genes showing differential transcript abundance between conditions with an adjusted p-value <= 0.05 and finally the subset of the latter showing differential transcript abundance with a fold change > 2. The analysis pipeline utilises a single configuration file describing the samples, the location of the sequence files and the conditions, and with one command converts duplicate-marked BAM files into the DeTCT pipeline output gene list tables. These tables show the closest strand-specific Ensembl gene to the TC 3′ end, the region called as a peak, the unadjusted p-value, the adjusted p-value, the fold change between conditions and the count data. It is presented in tab or comma-separated tables or as an HTML table (see Additional file 2 for an example of a tab-separated table).

### Data interpretation

The experimental rationale suggests that all transcript counting 3′ ends (TC 3′ ends) should match the 3′ ends of transcripts. However, gene annotation is sometimes incomplete and occasionally both the annotation and TC 3′ end can arise from experimental artefact. Therefore the DeTCT output specifies the distance between the TC 3′ end and the nearest Ensembl transcript 3′ end on the same strand. Naturally, this assumes the transcript to be annotated correctly and this is not necessarily true (see Additional file 3). The Ensembl transcript ends that exactly match the TC 3′ ends can be easily filtered from the results table. Non-exact matches suggest incomplete gene annotation or novel alternative transcript ends and both situations can be validated by individual inspection (see Additional file 4). Choosing a close proximity filter of the coordinates, such as between -100 bases (towards the 5′) and +100 bases (towards the 3′), reduces the likelihood of a false positive match and using these criteria we were able to detect a mean of 9664 genes per experiment (Fig. 2d). In contrast a more relaxed proximity filter, such as between -100 to +5000, identifies many more genes (mean 12750), but also finds more false positive ends. This is discussed further in the comparison to RNA-seq below. One cause of false positive TC 3′ ends is oligo dT priming from polyA or degenerate polyA sequence within RNA molecules or possibly from residual DNA. We identified falsely primed TC 3′ ends during the DeTCT analysis pipeline by examining the 10 bases 3′ of the TC 3′ end and removed those which potentially derived from non-polyA tail priming using the criteria described in the methods (see Additional file 4). One zebrafish-specific example is in the mitochondrial genome where a region rich in adenine (MT:2501-2518) in the rRNA ENSDARG00000080337 accounts for between 15 % to 23 % of the total counts in the four test experiments (Additional file 1). This example escaped our current polyA filtering method. To mitigate false positive 3′ end calls, we have begun to prepare a list of TC 3′ ends we believe not to be true transcript TC 3′ ends. Similarly, we are able to catalogue all the true positive TC 3′ ends we find and build cross-experiment profiles of regularly identified TC 3′ ends. We further filtered the results file by restricting the regions used to those with the 3′ coordinate of the region not more than 150 bases upstream of the TC 3′ end.

### Assessing the DeTCT method

**Fig. 3:**
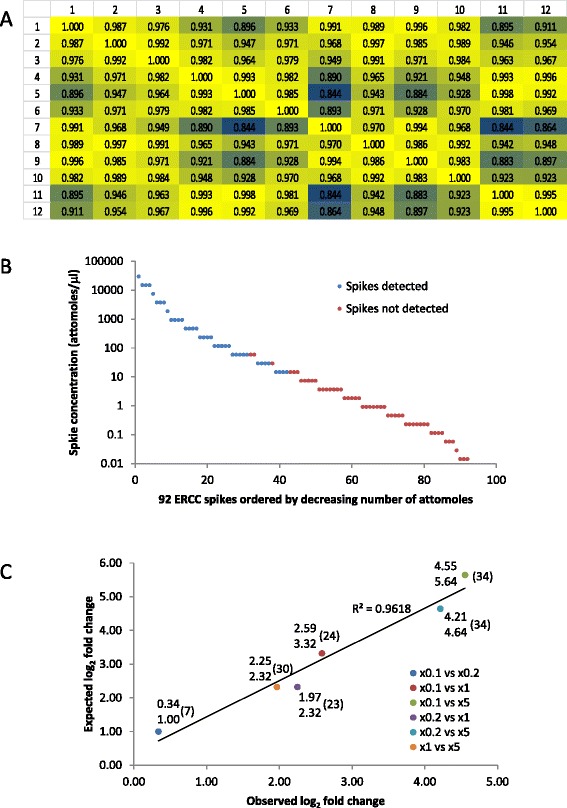
Technical replicate. Twelve replicate transcript counting libraries were prepared from 1 μg of a pool of wild-type zebrafish embryo total RNA sample. Libraries were sequenced by Illumina MiSeq and analysed using the DeTCT pipeline. **a** The normalised counts for each region were extracted (73,938 regions). After filtering the data for only the regions which we would use to call differential transcript abundance the counts from all 12 libraries were compared using a Pearson correlation (see Additional file 5). Cells coloured yellow in the Pearson correlation are the most highly correlated while those in blue are the least correlated with a colour gradient inbetween. **b** In addition four concentrations of ERCC spike mix 1 were added in triplicate to the same 12 libraries prior to library construction. We added the quantity suggested by the manufacturer (x1), five times the quantity (x5), one fifth of the quantity (x0.2) and one tenth of the quantity (x0.1). The reads were mapped to the zebrafish reference sequence and ERCC spike reference sequence. The diagram shows the 92 ERCC spikes represented by a circle in descending order of spike copy number in the mix on the x-axis and spike abundance on the y-axis. The blue circles show spikes identified in the DeTCT pipeline while those in red were not found. **c** The DeTCT analysis pipeline was run using six libraries at a time with three replicates as one pair of conditions and in all six possible condition combinations. The mean log_2_ fold change was calculated for all the spikes detected by the DeTCT analysis and plotted against the expected log_2_ fold change as circles. Each circle is labelled with the conditions being compared and observed log_2_ fold change over the expected log_2_ fold change. The numbers in brackets indicate how many spikes show differential transcript abundance

To assess the variation between libraries we extracted RNA from a pool of zebrafish embryos and made 12 replicate transcript counting libraries with different indexing sequences using 1 μg each. Libraries were pooled, sequenced on an Illumina MiSeq and analysed using the DeTCT pipeline. The number of normalised counts for each genomic region called by DeTCT for each library was determined. The regions were filtered for a maximum 100 bp distance to an Ensembl gene 3′ end, as described in Additional file 4, and compared using a Pearson correlation (Fig. 3). These 12 libraries show our method displays good technical reproducibility (the Pearson correlations of the unfiltered regions are shown in Additional file 5).

To examine the method’s performance in quantifying differential transcript abundance we added ERCC spike mix 1 (Ambion) to the total RNA prior to making the 12 technical replicate libraries described above. We added the quantity of spike mix recommended by the manufacturer, five times the quantity, a fifth of the quantity and a tenth of the quantity, all in triplicate, to create four conditions. After analysis through the DeTCT pipeline we identified 39 spikes (three spikes identified two TC 3′ ends). The spikes with high copy number in the mix were detected, but not those with lower copy number (Fig. 3b). The mean log_2_ fold changes were calculated for the spikes detected in all combinations of condition and compared to the log_2_ fold change expected (Fig. 3c). We found a good correlation between observed and expected log_2_ fold change. Additionally, in three of the pairwise comparisons no false positives were detected and in the other three only a total of six non-ERCC regions were found to be differentially expressed, suggesting that the method has high specificity.

**Fig. 4:**
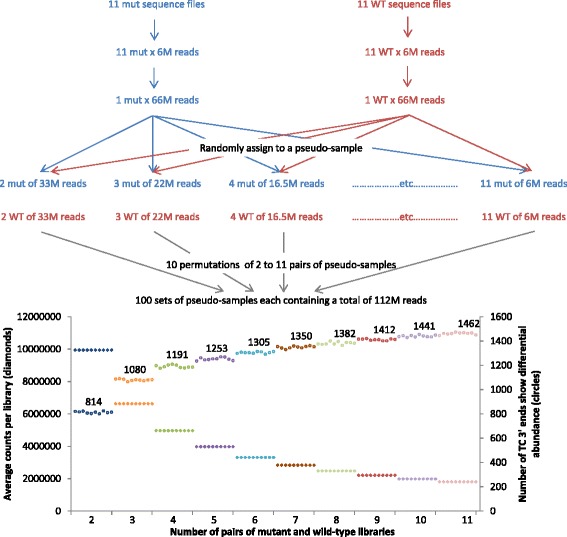
Sample number. The 11 mutant and 11 wild-type zmp_ph46 BAM files were downsampled and merged to create two BAM files, one from the mutant samples and one from the wild-type samples. The real index sequences in each BAM file were then replaced with fake index sequences in order to simulate assigning the reads at random to between 2 to 11 different pseudo-samples. Each of these 10 permutations was repeated 10 times, to give 100 simulations in total (shown on the x-axis), each of which was run through the DeTCT pipeline. The chart shows the average total number of counts for each pseudo-sample (diamonds on the left y-axis) and the number of (transcript counting 3′ end) TC 3′ ends with a relaxed Ensembl transcript proximity filter showing differential transcript abundance (circles on the right y-axis) with the mean above each group of ten

Our method was designed to maximise the number of tissue samples we can process with relatively shallow sequencing whilst still obtaining sufficient information to implicate gene networks modified by the condition change. The ability to make numerous transcript counting libraries has two main advantages. Firstly, problem libraries resulting from sample loss or showing low complexity can be excluded from analysis and their removal has little effect on the statistical power of the analysis. Similarly, samples with incorrectly assigned condition have less influence on the final result. Secondly, increasing the number of replicates improves the statistical power of the analysis. To assess the impact of increasing the number of replicates whilst retaining the same total amount of sequencing we performed a permutation test using the zmp_ph46 data [30]. Reads were combined to produce two samples comprising 66,000,000 mutant and 66,000,000 wild-type reads. These were randomly split ten times into ten collections of pseudo-samples each containing equal numbers of reads but with the number of pairs of pseudo-samples ranging from two pairs to 11 pairs (Fig. 4). After passing these collections of pseudo-samples through the DeTCT pipeline (see Fig. 4 for details) we identified 22,200 transcript counting 3′ ends (TC 3′ ends) in all 100 simulations. Although this simulation has removed the variance from the original biological replicates it shows that increasing the number of libraries at the expense of read depth improves detection of TC 3′ ends showing differential abundance, as was previously noted [31].

**Fig. 5:**
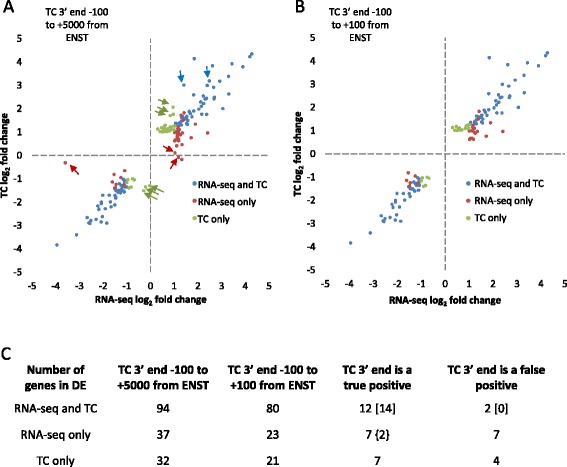
Comparing RNA-seq and transcript counting. The genes identified as showing differential transcript abundance in experiment *lamc1* ^*sa379*^ with an adjusted p-value <= 0.05 and an absolute fold change of >= 2 by one or both methods at the less stringent proximity filter between the transcript counting 3′ end (TC 3′ end) and the Ensembl transcript end (Table 1 row 16) were plotted with their log_2_ fold change from RNA-seq against the log_2_ fold change from TC. Genes with an adjusted p-value <= 0.05 and absolute fold change >= 2 in both RNA-seq and TC are shown as blue circles. Genes which fail on one or both criteria in RNA-seq but not in TC are shown as green circles and vice versa as red circles. **a** The log_2_ fold changes of genes with the TC 3′ end and Ensembl transcript proximity filter between -100 and +5000. The arrows highlight examples of genes which are not seen in graph B. **b** The log_2_ fold changes of genes with the proximity filter between -100 and +100. c A table showing the number of genes represented by each circle colour. The fourth and fifth columns show the genes which are lost during the more stringent proximity filtering and whether these are considered true positives or false positives after examining the TC 3′ end. Note that for two genes where the RNA-seq and TC data correlated, the closest TC 3′ end was found to be false, but a true end was identified further downstream and indicated by square brackets. The number in curly brackets indicates genes with a true TC 3′ end and an absolute fold change >2 but which fail to show an adjusted p-value <= 0.05 in TC

Many mRNA expression pipelines use whole transcript RNA-seq protocols and a range of analysis tools [32]. We don’t intend to replace these methods but present an alternative method for high-throughput mRNA expression screening. However, it is useful to compare the results of both methods. To this end we made DeTCT libraries and standard non-directional polyA pulldown Illumina RNA-seq libraries for two alleles. The same three wild-type and three mutant zebrafish total RNA samples were processed for each method, plus two or six additional libraries using the DeTCT protocol. We sequenced one HiSeq 2000 lane equivalent for each allele by each method (Table 1). Potential duplicate reads were identified and eliminated. The UMI in DeTCT allowed a more accurate identification of duplicates and hence fewer reads were dismissed as duplicates. Read 2s from the RNA-seq data that mapped to the genome were compared to Ensembl gene annotation to produce count data for each gene and the DeTCT pipeline was used to extract count data linked to Ensembl transcripts from the transcript counting reads. DESeq2 was run on both sets of count data. Even after removing duplicate reads there are generally more counts in the RNA-seq count data (Table 1). This is probably due to a drop in read quality following the oligo dT sequence in transcript counting (TC) read 1. RNA-seq initially identifies more genes, however, the gap between the methods is reduced substantially when regions with a low mean count (which are unlikely to be called significantly differentially expressed due to lack of power) are filtered out by DESeq2 (Table 1 row 11). The number of TC 3′ ends (Table 1 row 9) is higher than that of DeTCT genes (Table 1 row 8) which suggests alternative 3′ ends [6, 33] (see Additional file 4 for an example). However, some represent false positive TC 3′ ends which escape our filter for false oligo dT priming. To assess the genes showing differential transcript abundance the full gene list was filtered for protein-coding genes with an adjusted p-value of <= 0.05 and an absolute fold change >= 2 (log_2_ fold change <-1 or >1) between mutant and wild-type (Table 1 row 13 and volcano plots in Additional file 6). RNA-seq identifies more genes, but using a less stringent proximity filter between the TC 3′ end and the 3′ end of an Ensembl transcript increases the detection rate in DeTCT. For a direct comparison between the two methods we identified the genes with an adjusted p-value <= 0.05 and absolute fold change >= 2 (Table 1 row 16) for both methods and applied the less stringent proximity filter to the TC 3′ ends. The fold change for these genes in RNA-seq and TC was compared (Fig. 5 for *lamc1*^*sa379*^ and Additional file 7 for *mdn1*^*sa1349*^) and showed a good correlation with r^2^ = 0.96 (blue circles on Fig. 5a), which suggests the two methods are finding the same alterations in transcript abundance. We next looked at genes which show an adjusted p-value <= 0.05 and an absolute fold change >= 2 by one method, but failed to meet one or both criteria by the other (red and green circles on Fig. 5a). For both methods 14 genes have an absolute fold change >= 2, but fail to have sufficient power to call an adjusted p-value <= 0.05 by one method. Similarly, for 38 genes where one method fails to show an absolute fold change >= 2 the actual fold change is just below 2 (cut off log_2_ fold change >= 0.8 or <= -0.8) suggesting further genes where the two methods give comparable fold change results. We then applied the stringent TC 3′ end proximity filter to the same data which led to the removal of 39 genes (Fig. 5b). Examining the TC 3′ end of these 39 genes showed they fell into two groups, either true TC 3′ ends or false TC 3′ ends assumed to be derived from experimental artefact (Fig. 5c). Where both methods gave an adjusted p-value <= 0.05 and an absolute fold change >= 2 all 14 were shown to be true ends (note that in two cases the closest TC 3′ end was found to be false, but a true TC 3′ end was found downstream). By contrast, in the gene sets only called by one method 11/25 TC 3′ ends lost to more stringent filtering were false positives. Together this analysis shows the removal of 39 genes by increasing the stringency of the DeTCT proximity filter resulted in losing 28 true positives (14 were only found by one method), but prevented calling 11 false positive TC 3′ ends.

**Table 1:** Comparison of RNA-seq and transcript counting

		*lamc1* ^*sa379*^			*mdn1* ^*sa1349*^		
	Library protocol	RNA-seq	TC		RNA-seq	TC	
	Number of libraries	x6	x12		x6	x8	
1	Number of read 2s mapped	146M	84M		119M	97M	
2	Reads removed as duplicates	31.4 %	11.7 %		25.3 %	9.8 %	
3	Counts (map to ENSG or called as peaks)	67M	52M		61M	61M	
4	Number of counts matching MT transcripts	2.25M	5.51M		1.23M	3.73M	
5	Proximity TC 3′ end and Ensembl transcript in bases	NA	−100 to 5000	−100 to +100	NA	−100 to 5000	−100 to +100
6	Number of counts calling genes (no MT)	65M	21M	14M	60M	24M	19M
7	[Number of counts used to call transcripts]	-	27M	16M	-	31M	20M
8	Number of genes detected (no MT)	27732	14544	9906	28455	17138	11419
9	[Number of transcripts detected]	-	21220	10574	-	25034	12144
10	Protein-coding genes with pval obtained	24256	13763	9542	25012	16220	10967
11	Protein-coding genes with adj pval obtained	15883	12139	8555	22034	14886	10184
12	And with an adj pval <=0.05	1468	227	162	9529	2649	2013
13	And fold change >=2	235	141	103	2255	1534	1121
14	Genes identified by RNA-seq and TC						
15	Genes from row 10 identified by both	11114	11114		14791	14791	
16	And with an adj pval <=0.05 and FC >2	131	126		1443	1427

## Conclusion

We present a quantitative mRNA transcript profiling package that starts with tissue samples and produces a gene list by counting the 3′ end of any polyadenylated transcripts using Illumina sequencing. Unlike whole transcript RNA-seq each transcript is counted only once giving a more representative estimate of transcript abundance [24]. Short, rare transcripts are as likely to be represented in the sequence as long, rare transcripts. Assaying only the 3′ end of transcripts is also more resilient to degraded RNA samples, particularly if cells or tissues are compromised by a treatment. Differential transcript abundance is identified based on genome sequence and is independent of gene annotation. This highlights regions containing novel transcripts as well as previously undescribed alternative transcript 3′ ends, which are implicated in biological processes related to the condition. Each alternative transcript 3′ end is represented by discrete count data and has the potential to add layers of functional annotation to sequence at the 3′ ends of gene models.

Our streamlined library preparation reduces material loss and allows us to produce large numbers of libraries. Working with more replicates not only provides more power to the differential expression calculation but also lessens the impact of occasional sample loss or failure. This allows us to measure transcriptional changes even in rare tissues within the whole organism. The addition of a unique molecular identifier (UMI) helps us to assess the quality of each library preparation and we can remove any which underperform from the analysis. Simultaneously processing a large number of samples allows us to ask complex questions, such as what is the impact of different concentrations of a compound at varying stages of embryonic development? Similarly, we could screen large numbers of mutagenised individuals following an infection challenge to identify variation in infection response and inflammation. In both cases relatively shallow sequencing of each sample will result in reduced sensitivity, but can highlight a critical combination of conditions to explore further using deeper sequencing of selected libraries to increase the number of transcripts detected or by an alternative method such as RNA-seq. We are also able to assay large numbers of wild-type samples producing a gene profile baseline that can be refined with data from each individual biological replicate. For precious or difficult to obtain RNA samples conventional full transcript RNA-seq methods are more suited, but for rapid assessment of many samples with a desire to implicate a molecular process, transcript counting is the better option. The gene lists provided by DeTCT are the link between a living organism and the array of gene ontology, gene expression and gene interaction network data in the public domain. We believe the power of the differential expression transcript counting technique (DeTCT) lies in effectively and efficiently bridging this gap.

## Methods

### Sequence data submission

[EMBL:E-ERAD-244, EMBL:E_ERAD-121, EMBL:E-ERAD-91, EMBL:E-ERAD-384].

### Sample collection

Breeding zebrafish (*Danio rerio*) were maintained at 28.5 °C on 14h light/10h dark cycle. Fertilised eggs were obtained from pairs of heterozygous fish carrying nonsense mutations in transcripts of specific genes [16] by natural spawning. They were then grown in incubators at 28 °C (except samples prefixed with zmp_ph9 which were grown at 32 °C), separated into morphologically abnormal and morphologically normal sibling embryos at the correct developmental stage and snap frozen in dry ice or liquid nitrogen. Over the course of the protocol development we have refined the RNA extraction protocol to allow increased sample throughput. RNA was extracted for samples prefixed *lamc1*^*sa379*^, *mdn1*^*sa1349*^, zmp_ph35 and zmp_ph40 by lysis in Trizol (Invitrogen). The lysate was mixed with 0.2 volumes of chloroform and processed in Phase Lock Gel heavy 2 ml tubes (5 Prime) according to the manufacturer’s instructions. For *lamc1*^*sa379*^ and *mdn1*^*sa1349*^ the aqueous phase was transferred to an RNase free 1.5ml tube (Ambion) and precipitated using 0.5 ml isopropyl alcohol per 1 ml Trizol reagent used. The samples were then spun at 2–8 °C for 10 min at 12,000 rpm. The supernatant was discarded and the pellet was washed twice with 75 % ethanol. The pellet was then dried at room temperature for 10 min and dissolved in 30 μl of RNAse free water. RNA was quantified using a Nanodrop. For samples zmp_ph35 and zmp_ph40 the aqueous phase from the phase lock column was collected, mixed with equal volumes of 70 % ethanol and applied to an RNeasy MinElute column (Qiagen). The columns were spun for 15 s at 8000 x g at room temperature in a centrifuge. The columns were then washed with 500 μl of RPE buffer (Qiagen) followed by 500 μl of 80 % ethanol. After drying the columns at full speed in a centrifuge, RNA was eluted from them in 16 μl of nuclease free water. RNA was quantified using Qubit RNA HS assay (Invitrogen). For samples zmp_ph45 and zmp_ph46 the embryos were lysed in 100 μl of RLT buffer (Qiagen) containing 1 μl of 14.3M beta mercaptoethanol (Sigma) in 2ml RNAse free tubes. The lysate was mixed with 1.8 volumes of Agencourt RNAClean XP (Beckman Coulter) beads, mixed by pipetting and allowed to bind for 10 min. The tubes were then applied to a magnetic rack (Invitrogen) until the solution turned clear and the supernatant was removed without disturbing the beads. While still on the magnet, the beads were washed three times with 70 % ethanol and air dried for 10 mins. The beads were re-suspended in 50 μl of RNAse free water by pipetting. The RNA was then eluted from the beads by applying the tubes to the magnetic rack. RNA was quantified using Qubit RNA HS assay (Invitrogen).

### Library preparation and sequencing

Libraries for zmp_ph35, zmp_ph40, zmp_ph45 and zmp_ph46 were made in 96 well plates. DNA was removed from 300 ng of total RNA by treatment with 2 units of RNase-Free DNase I (NEB) in 100 μl reaction using the manufacturer’s buffer for 10 min at 37 °C and heated to 75 °C for 90 min to fragment the RNA. For each library 12 μl of Streptavidin magnetic beads were washed in 1x wash/binding buffer (20 mM Tris pH 7.5, 0.5 M NaCl, 1 mM EDTA) and 1 μl of 10 μM biotinylated polyT primer (B-TAATGCGGCCGCABCBTBTCAGTCTTTTTTTTTTTTTTVN (note the sequence 5′ of the polyT including the *Not*I site is not required for this protocol and an anchor polyT_30_ will suffice) was added. The primer was bound with rotation for 5 min at room temperature and the beads washed. After adding an equal volume of 2x wash/binding buffer and 40 units of RNase Inhibitor (NEB) the cold RNA was added and allowed to bind for 20 min at room temperature with rotation. The beads were washed twice in 1x wash/binding buffer, once in cold low salt buffer (0.15 M NaCl, 20 mM Tris-HCl pH 7.5) and suspended in water. The RNA was phosphorylated with 1 unit of T4 Polynucleotide Kinase (3′ phosphatase minus) (NEB) for 30 min at 37 °C with 40 units of RNase Inhibitor and the RNA oligo stRSSA4 (5′ Am-CUCGGCAUUCCUGCUGAACCGCUCUUCCGAUCU; all Illumina adapter sequences are from [34]) ligated with 20 units of T4 RNA ligase (NEB) in the presence of 20 % PEG 8000 (Promega) for 120 min at 37 °C. After adding an equal volume of 2x wash/binding buffer and incubating at room temperature for 2 min the beads were washed twice in 1x wash/binding buffer, once in cold low salt buffer and re-suspended in water. The RNA was eluted from the beads by heating at 80 °C for 2 min and separated on a magnet. One of 96 indexed primers (8mer_SC_TC 1 to 96 - ACACTCTTTCCCTACACGACGCTCTTCCGATCTNNNNBBBBNNNNXXXXXXXXCGTTTTTTTTTTTTTTVN - generic primer where X represents an 8 base index as described in Additional file 1, N is A, C, G or T, B is C, G or T and V is A, C or G) was added to each RNA sample (1 μl of 10 μM), then heated to 70 °C and snap chilled on ice. Reverse transcription was performed using SuperScript II (Invitrogen) in the presence of 40 units of RNase Inhibitor according to the manufacturer’s instructions, followed by the addition of 1 unit of Exo1 (NEB), incubated at 37 °C for 30 min and then 80 °C for 20 min and finally cleaned with the QIAgen PCR clean-up kit. Libraries were amplified to complete the Illumina adapter sequence using SAPCRS. 1 (5′-AATGATACGGCGACCACCGAGATCTACACTCTTTCCCTACACGA-3′) and SAPCRS. 2 (5′-CAAGCAGAAGACGGCATACGAGATCGGTCTCGGCATTCCTGCTGAAC-3′) in a 50 μl reaction containing 35 μl of library, 5 μl of 10X KOD buffer, 5 μl of 2mM dNTPs, 2 μl MgSO_4_ , 2 μl of 10 μM primer mix and 1 μl of KOD HOT start polymerase (Novagen) by incubating in a pre-heated DNA Engine Tetrad (MJ Research) at 94 °C for 2 min, then 94 °C for 15 s, 60 °C for 30 s and 68 °C for 3 min for 20 cycles and finishing with 68 °C for 5 min. Libraries were cleaned with the QIAgen PCR clean-up kit, quantified using a BioPhotometer (Eppendorf), mixed in equimolar quantities, size selected with Spri beads for an insert size of 70-270 bases and quantified by qPCR. Sequencing was performed on an Illumina HiSeq 2500. The *lamc1*^*sa379*^ and *mdn1*^*sa1349*^ libraries were made in 1.5 ml RNase free tubes (Ambion) using an earlier protocol which is the same as that described above for zmp_ph35 except 5 μg of total RNA from pools of embryos were used for each library, total RNA was treated with DNase for 10 min at 37 °C followed by inactivation at 75 °C for 10 min in EDTA, then ethanol precipitated, fragmented with Ambion fragmentation reagent for 5 min at 70 °C, pulled down with 62.5 μl of streptavidin beads, the reverse transcription primer contained the sequence NNNNB instead of NNNNBBBBNNNN, there was no ExoI digestion step and only 15 cycles of amplification. The libraries were sequenced on HiSeq 2000.

### DeTCT analysis pipeline

The source code for the DeTCT pipeline is available from DeTCT github [35]. Prior to running the DeTCT pipeline, the sequencing reads were processed with the detag_fastq.pl script, which trimmed reads to improve quality and rejected read pairs where the first read of the pair did not begin with the unique molecular identifier (UMI), followed by a sample specific index sequence and polyT. These sequences were removed from the read, and the index and UMI were added to the read name. The reads were aligned to the Zv9 zebrafish reference genome [36] with BWA 0.5.10 [37] and converted to BAM format with SAMtools [38]. The resulting BAM files were processed with Picard MarkDuplicates [26] to fix mate information and add read groups. Duplicate reads were identified using a modified version of Picard’s MarkDuplicates called Picard-detct [26, 27], which took into account the UMI in the read name. The final BAM files were used as input for the DeTCT pipeline. In the first stage, an HMM-based peak caller, HPeak [39], was used to identify regions where read 2 of each read pair was aligned. All the second reads in an experiment were put into 100 bp bins, with duplicate reads and reads with more than 2 mismatches being ignored, and these bins were used as input to HPeak. The output is the probability that each bin represents a peak, with adjacent bins being merged to create regions. For each region, all the read 2s aligned in that region were identified and then the read 1 associated with each read 2 was determined. Due to the library construction method, the alignment of read 1 marks the 3′ end of a transcript. Transcript counting 3′ ends (TC 3′ ends) were ignored if they were supported by fewer than 3 reads or if the 10 bp sequence downstream was significantly enriched in A bases (4 As at the start or more than 6 As in total or matching one of the following empirically determined patterns: AAABAAABBB, AAABAABABB, AAABABAABB, AABAAAABBB, AABAAABABB, AABABAAABB, ABAAAAABBB, ABAAAABABB, ABAAABAABB, ABAABAAABB, ABABAAAABB, AABAABAABB). The TC 3′ end with the highest read count was associated with each region. Finally, the number of read 2s aligned in each region was determined for each sample and these counts were used for differential expression analysis using DESeq2 [28]. All regions with a TC 3′ end were associated with Ensembl gene annotation based on the nearest transcript in the appropriate direction on the correct strand. The final output was a table (in CSV, TSV or HTML formats) containing region coordinates, associated TC 3′ end coordinates and read counts, differential expression p-value and adjusted p-value, gene and transcript annotation, distance of TC 3′ end to nearest Ensembl 3′ end, count data, normalised count data and log_2_ fold changes.

### Technical replicate

Twelve transcript counting libraries were prepared using 1 μg of total RNA extracted from a pool of zebrafish embryos. As recommended by the manufacturer for 1 μg of total RNA a 1:100 dilution of spike mix 1 (Ambion) was made and 2 μl added to the three x1 samples. We added 5 times this quantity to the x5, one fifth to the x0.2 and one tenth to the x0.1. Sequencing was performed on an Illumina MiSeq. The reads were aligned to the Zv9 reference genome for the technical replicate and to the Zv9 reference genome including the spike reference sequences for the differential abundance test. For the technical replicate the duplicate-flagged sequence was passed through the DeTCT pipeline, the results filtered as described in additional file 5 and the resulting normalised count data compared by calculating Pearson’s product moment correlation coefficient using R’s cor.test function. For the differential abundance test the DeTCT pipeline was run on all 12 samples to get count data in triplicate for the four conditions (x5, x1, x0.2, x0.1). Then all six pairwise comparisons of the four conditions were run from the DESeq2 step onwards with the relevant six libraries. The mean of the log_2_ fold change of the spikes was calculated and compared to the expected log_2_ fold change.

### Sample number

The 11 mutant and 11 wild-type zmp_ph46 BAM files were downsampled to 6 million read pairs each using the downsampling pipeline of the DeTCT pipeline [35] and then merged using Picard [26] to create two BAM files, one containing 66 million read pairs from the 11 mutant samples and one containing 66 million read pairs from the 11 wild-type samples. The real index sequences in each BAM file were then replaced with fake index sequences using Pseudo bam files [40] in order to simulate assigning the reads at random to between 2 to 11 different pseudo-samples. Each of these 10 permutations was repeated 10 times, to give 100 simulations in total. Each simulation was then run through the DeTCT pipeline.

### Comparison of RNA-seq and transcript counting

For two different knockout alleles we made six non-directional TruSeq PE Cluster Kit v3 RNA-seq and six TC libraries from three wild-type and three mutant zebrafish total RNA samples, plus two or six additional libraries using the TC protocol (Table 1). Libraries were sequenced using paired-end 75 bp Illumina HiSeq 2000 systems, with reads trimmed to 54 bp using the DeTCT pipeline or the FASTX-Toolkit. Read 2s were mapped to the Zv9 reference genome with BWA 0.5.10 (row 1). Duplicate reads were identified (row 2) using the modified version of Picard’s MarkDuplicates called Picard-detct [26, 27]. RNA-seq read counts for read 2 were obtained with htseq-count using the Ensembl 74 annotation, whilst the DeTCT pipeline was used to extract TC read counts (row 3). Counts mapped to the mitochondrial genome were excluded from further analysis (row 4). The proximity of the Transcript counting 3′ end (TC 3′ end) and an Ensembl transcript were filtered at a high stringency (between -100 and +100 bases) or low stringency (between -100 and +5000 bases) (row 5). For the RNA-seq analysis all counts match a gene (rows 6 and 8), whereas in the TC analysis only counts where the TC 3′ end is associated with an Ensembl gene (rows 6 and 8) or transcripts (rows 8 and 10) are used and the remainder represent un-annotated genes, alternative 3′ ends or experimental artefact. In order to ensure a one to one correspondence between RNA-seq genes and TC genes, 28 Ensembl v74 transcripts thought to be fallacious were added to a blacklist [41]. Transcripts on the blacklist were not used to assign TC 3′ ends to Ensembl genes, ensuring a single gene was not annotated to different TC 3′ ends. In addition one RNA-seq read matching an exon in a gene is sufficient to call a gene, whereas multiple reads are required to call a TC 3′ end in DeTCT. DESeq2 was run on both sets of count data. Only protein-coding genes where a p-value was identified by DESeq2 were considered further (row 10) and from these a subset was also awarded an adjusted p-value (row 11). Genes showing differential transcript abundance between mutant and wild type with an adjusted p-value <= 0.05 (row 12) were further filtered for those with a fold change >= 2 (row 13). Before the comparison of the RNA-seq and TC methods the number of protein-coding genes with an adjusted p-value called by both methods (row 15) and the number showing an adjusted p-value <= 0.05 plus a fold change >= 2 (row 16) were identified.

### Ethical statement

Zebrafish were maintained in accordance with UK Home Office regulations, UK Animals (Scientific Procedures) Act 1986, under project licence 70/7606, which was reviewed by the Wellcome Trust Sanger Institute Ethical Review Committee.

## Additional files

Additional file 1:
**Table of libraries, ENA accession, sequence depth and indexing sequence.** (XLSX 22 kb) Additional file 2:
**Example of a DeTCT output table.** An example of a DeTCT output table showing the column header and 54 rows of regions with the adjusted p-value of <=0.05 for zmp_ph45 (allele *pla2g12b* ^*sa659*^) and ordered from the lowest adjusted p-value. The table is not filtered for proximity of TC 3′ end to the nearest Ensembl gene (see column 9). (XLSX 33 kb) Additional file 3:
**TC 3′ end of unannotated transcript.** Screen shots from the forward strand of Ensembl version 75 browser are shown. A. Region 10:40424001-40469000 configured with the following Genes and Transcript tracks: Pooled RNA-seq (blue gene models), Pooled RNA-seq alignments (grey bars), intron tracks for 2 cell, 1 dpf and 14 dpf (blue/green bars) and the merged Ensembl/Havana gene model ENSDART00000055339 (dark red). An additional transcript was identified during the gene build using RNA-seq data (RNASEQT00000024319 – marked by a red arrow) which was filtered from the final transcript set [29]. B. The region at the 3′ end of ENSDART00000055339 is expanded to show the TC 3′ end at coordinate 10:40464260 (red circle), which is 448 bases 5′ of the annotated end. RNA-seq data supports the TC 3′ end but not the final exon of the annotated transcript. C. The region containing RNASEQT00000024319 is expanded to show the TC 3′ end at co-ordinate 10:40428902 (red circle), which is 35,806 bases upstream of the closest strand-specific transcript 3′ end of ENSDART00000055339 and in the initial DeTCT output tables this coordinate is associated with the annotated transcript by the DeTCT pipeline. Filtering for proximity between the TC 3′ end and the 3′ end of the nearest annotated transcript at +/- 100 bases or -100 to +5000 bases removes the link between coordinate 10:40428902 and the adjacent transcript avoiding a false positive call. Future Ensembl gene builds will hopefully identify the missing transcript. (PDF 50 kb) Additional file 4:
**Alternative transcript counting 3′ ends in 3′ UTR.** Screenshots from the forward strand of Ensembl version 75 browser are shown. A. Region 6:10360000-10500000 configured with the following Genes and Transcript tracks: Pooled RNA-seq alignments (grey bars) and the merged Ensembl/Havana gene model ENSDARG00000062687 (dark red). B. The 9 kbp region at the 3′ end of the ENSDARG00000062687 gene model. C to F. Details of the four regions identified by the DeTCT pipeline. Red circles indicate the genomic coordinate of the TC 3′ end. The TC 3′ end at 6:10493860 (panel F) shows evidence of a polyadenylation signal and no genomic polyA track supporting it being a true transcript 3′ end but is 6256 bases downstream of the Ensembl gene model 3′ end. The TC 3′ end at 6:10489461 (panel C) may be an alternative transcript 3′ end, but could have arisen from priming off the surrounding polyA tracts. The other two TC 3′ ends (D and E) have less evidence and may have arisen by priming off the local polyA tracts. (PDF 68 kb) Additional file 5:
**Technical replicate.** Twelve replicate transcript counting libraries were prepared from 1 μg of a pool of wild-type zebrafish embryo total RNA sample. The libraries were sequenced by Illumina MiSeq and analysed using the DeTCT pipeline. The normalised counts for each region were extracted (73,938 regions). A. The coefficient of variance was calculated for all regions and plotted against the mean of the counts (blue, red and green circles) and the Pearson correlations shown in part B. Regions with a low mean count (i.e. with little or no chance of showing significant differential expression) were removed using DeSeq2 independent filter and the remaining 7,379 regions plotted on the same graph (red and green circles) and the Pearson correlations shown in part C. The proximity of the transcript counting 3′ ends (TC 3′ ends) was restricted to within 100 bases of an Ensembl transcript 3′ end and the resulting 1,976 regions plotted on the same graph (green circles) and the Pearson correlations shown in Fig. 3. The graph shows less dispersion between the filtered regions of the 12 technical replicates compared to the unfiltered regions. It was noted that one region mapping to the mitochondrial MT:2501-2518 (within ENSDARG00000080337) comprised a large proportion of counts, distorting the Pearson correlation (red arrow on A). We believe these are derived by priming from a polyA sequence in the mitochondrial rRNA sequence. The Pearson correlation of all regions with this outlier removed is shown in part D and after removing regions with low mean counts is shown in part E. Note this mitochondrial region was removed in the Ensembl transcript proximity filter and therefore does not appear in the Pearson correlation shown in Fig. 3. Cells coloured yellow in the Pearson correlation are the most highly correlated while those in blue are the least correlated. The yellow to blue gradient is specific to each individual Pearson correlation. (PDF 165 kb) Additional file 6:
**Volcano plots of RNA-seq data.** Volcano plots, plotting the adjusted p-value against the log_2_ fold change, are shown for the two knockout alleles analysed by RNA-seq and TC. All transcripts with an adjusted p-value <= 0.05 are shown. Transcripts with a fold change >= 2 are blue and a fold change < 2 are red. TC transcripts were passed through the relaxed proximity filter of -100 to +5000. (PDF 127 kb) Additional file 7:
**Comparing RNA-seq and transcript counting.** The same analysis as Fig. 5, but using *mdn1* ^*sa1349*^. (PDF 97 kb)

## Abbreviations

DeTCT: differential expression transcript counting technique
TC 3′ end: transcript counting 3′ end
TC: transcript counting
UMI: unique molecular identifier
ZMP: zebrafish mutation project
