# Supplementary figures and images for: High-throughput and quantitative genome-wide messenger RNA sequencing for molecular phenotyping
Source: BMC Genomics. 2015 Aug 5;16(1):578. doi: 10.1186/s12864-015-1788-6 (PMC4524448; doi:10.1186/s12864-015-1788-6)

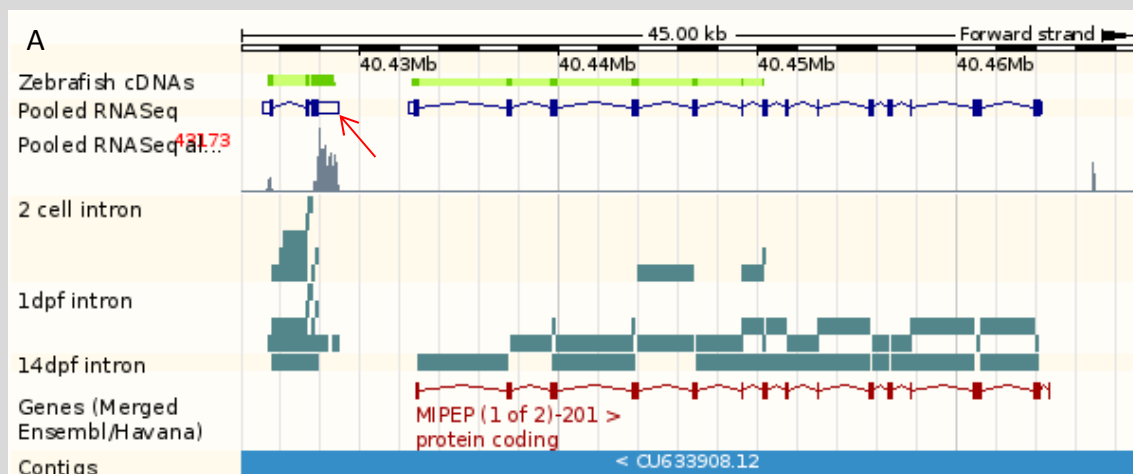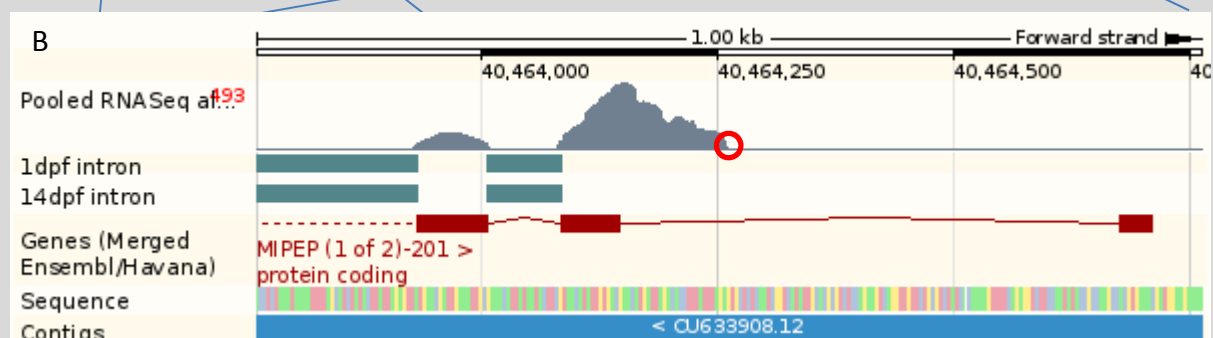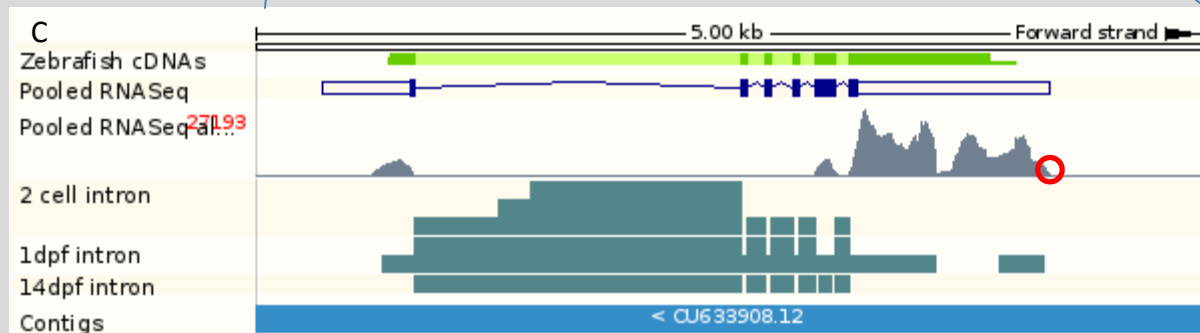

Supplement: Additional file 3: — TC 3′ end of unannotated transcript. Screen shots from the forward strand of Ensembl version 75 browser are shown. A. Region 10:40424001-40469000 configured with the following Genes and Transcript tracks: Pooled RNA-seq (blue gene models), Pooled RNA-seq alignments (grey bars), intron tracks for 2 cell, 1 dpf and 14 dpf (blue/green bars) and the merged Ensembl/Havana gene model ENSDART00000055339 (dark red). An additional transcript was identified during the gene build using RNA-seq data (RNASEQT00000024319 – marked by a red arrow) which was filtered from the final transcript set [29]. B. The region at the 3′ end of ENSDART00000055339 is expanded to show the TC 3′ end at coordinate 10:40464260 (red circle), which is 448 bases 5′ of the annotated end. RNA-seq data supports the TC 3′ end but not the final exon of the annotated transcript. C. The region containing RNASEQT00000024319 is expanded to show the TC 3′ end at co-ordinate 10:40428902 (red circle), which is 35,806 bases upstream of the closest strand-specific transcript 3′ end of ENSDART00000055339 and in the initial DeTCT output tables this coordinate is associated with the annotated transcript by the DeTCT pipeline. Filtering for proximity between the TC 3′ end and the 3′ end of the nearest annotated transcript at +/- 100 bases or -100 to +5000 bases removes the link between coordinate 10:40428902 and the adjacent transcript avoiding a false positive call. Future Ensembl gene builds will hopefully identify the missing transcript. (PDF 50 kb) [file 12864_2015_1788_MOESM3_ESM.pdf]

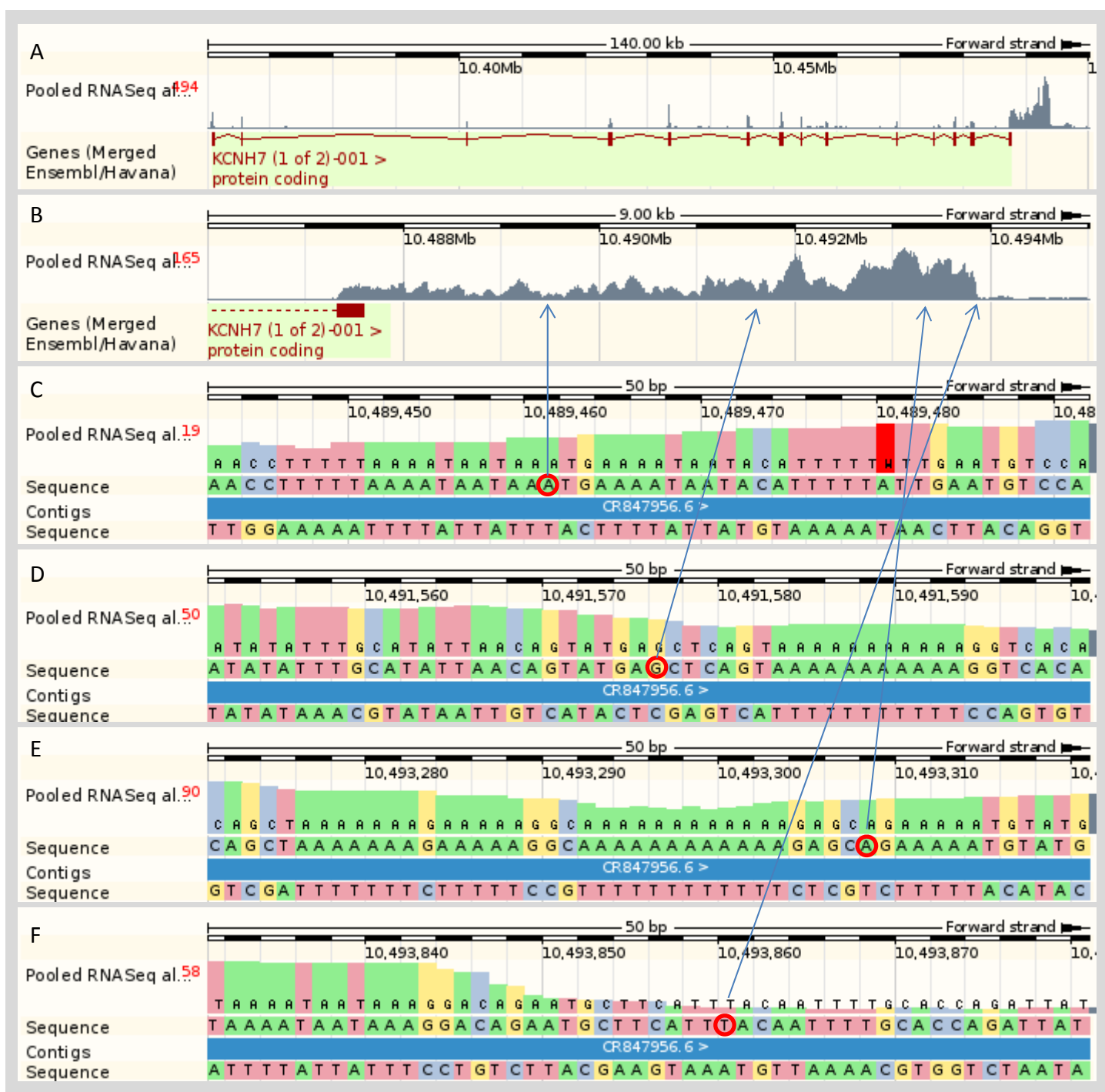

Supplement: Additional file 4: — Alternative transcript counting 3′ ends in 3′ UTR. Screenshots from the forward strand of Ensembl version 75 browser are shown. A. Region 6:10360000-10500000 configured with the following Genes and Transcript tracks: Pooled RNA-seq alignments (grey bars) and the merged Ensembl/Havana gene model ENSDARG00000062687 (dark red). B. The 9 kbp region at the 3′ end of the ENSDARG00000062687 gene model. C to F. Details of the four regions identified by the DeTCT pipeline. Red circles indicate the genomic coordinate of the TC 3′ end. The TC 3′ end at 6:10493860 (panel F) shows evidence of a polyadenylation signal and no genomic polyA track supporting it being a true transcript 3′ end but is 6256 bases downstream of the Ensembl gene model 3′ end. The TC 3′ end at 6:10489461 (panel C) may be an alternative transcript 3′ end, but could have arisen from priming off the surrounding polyA tracts. The other two TC 3′ ends (D and E) have less evidence and may have arisen by priming off the local polyA tracts. (PDF 68 kb) [file 12864_2015_1788_MOESM4_ESM.pdf]

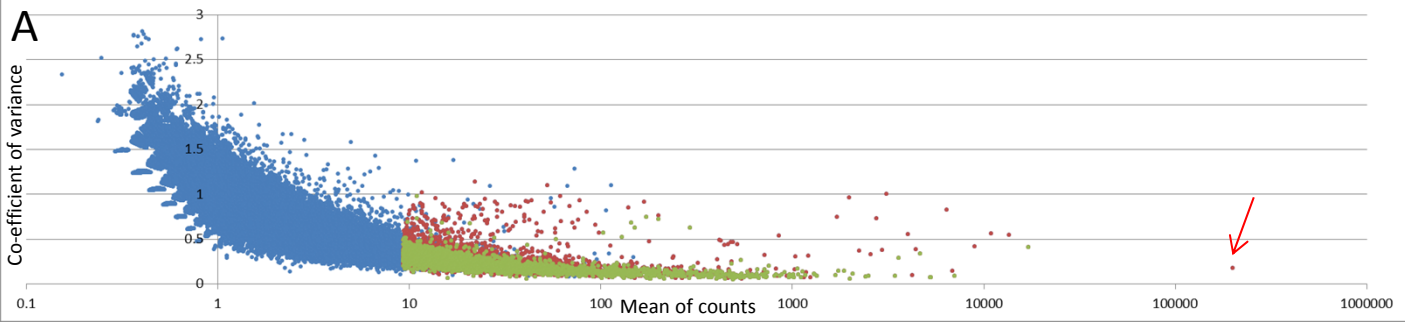

**B**

|    | 1     | 2     | 3     | 4     | 5     | 6     | 7     | 8     | 9     | 10    | 11    | 12    |
|----|-------|-------|-------|-------|-------|-------|-------|-------|-------|-------|-------|-------|
| 1  | 1.000 | 0.999 | 0.996 | 0.985 | 0.997 | 0.998 | 0.999 | 1.000 | 0.999 | 1.000 | 0.987 | 0.988 |
| 2  | 0.999 | 1.000 | 0.996 | 0.988 | 0.998 | 0.999 | 0.999 | 0.999 | 1.000 | 1.000 | 0.988 | 0.987 |
| 3  | 0.996 | 0.996 | 1.000 | 0.993 | 0.998 | 0.997 | 0.996 | 0.997 | 0.995 | 0.995 | 0.993 | 0.993 |
| 4  | 0.985 | 0.988 | 0.993 | 1.000 | 0.992 | 0.990 | 0.987 | 0.989 | 0.987 | 0.986 | 0.996 | 0.989 |
| 5  | 0.997 | 0.998 | 0.998 | 0.992 | 1.000 | 0.999 | 0.996 | 0.999 | 0.997 | 0.997 | 0.994 | 0.995 |
| 6  | 0.998 | 0.999 | 0.997 | 0.990 | 0.999 | 1.000 | 0.998 | 0.999 | 0.999 | 0.999 | 0.991 | 0.990 |
| 7  | 0.999 | 0.999 | 0.996 | 0.987 | 0.996 | 0.998 | 1.000 | 0.999 | 0.999 | 0.999 | 0.987 | 0.986 |
| 8  | 1.000 | 0.999 | 0.997 | 0.989 | 0.999 | 0.999 | 0.999 | 1.000 | 0.999 | 0.999 | 0.990 | 0.991 |
| 9  | 0.999 | 1.000 | 0.995 | 0.987 | 0.997 | 0.999 | 0.999 | 0.999 | 1.000 | 1.000 | 0.985 | 0.985 |
| 10 | 1.000 | 1.000 | 0.995 | 0.986 | 0.997 | 0.999 | 0.999 | 0.999 | 1.000 | 1.000 | 0.987 | 0.987 |
| 11 | 0.987 | 0.988 | 0.993 | 0.996 | 0.994 | 0.991 | 0.987 | 0.990 | 0.985 | 0.987 | 1.000 | 0.995 |
| 12 | 0.988 | 0.987 | 0.993 | 0.989 | 0.995 | 0.990 | 0.986 | 0.991 | 0.985 | 0.987 | 0.995 | 1.000 |

**C**

|    | 1     | 2     | 3     | 4     | 5     | 6     | 7     | 8     | 9     | 10    | 11    | 12    |
|----|-------|-------|-------|-------|-------|-------|-------|-------|-------|-------|-------|-------|
| 1  | 1.000 | 0.999 | 0.996 | 0.985 | 0.997 | 0.998 | 0.999 | 1.000 | 0.999 | 1.000 | 0.987 | 0.988 |
| 2  | 0.999 | 1.000 | 0.996 | 0.988 | 0.998 | 0.999 | 0.999 | 0.999 | 1.000 | 1.000 | 0.988 | 0.987 |
| 3  | 0.996 | 0.996 | 1.000 | 0.993 | 0.998 | 0.997 | 0.996 | 0.997 | 0.995 | 0.995 | 0.993 | 0.993 |
| 4  | 0.985 | 0.988 | 0.993 | 1.000 | 0.992 | 0.991 | 0.987 | 0.989 | 0.987 | 0.986 | 0.996 | 0.989 |
| 5  | 0.997 | 0.998 | 0.998 | 0.992 | 1.000 | 0.999 | 0.996 | 0.999 | 0.997 | 0.997 | 0.994 | 0.995 |
| 6  | 0.998 | 0.999 | 0.997 | 0.991 | 0.999 | 1.000 | 0.998 | 0.999 | 0.999 | 0.999 | 0.991 | 0.991 |
| 7  | 0.999 | 0.999 | 0.996 | 0.987 | 0.996 | 0.998 | 1.000 | 0.999 | 0.999 | 0.999 | 0.987 | 0.986 |
| 8  | 1.000 | 0.999 | 0.997 | 0.989 | 0.999 | 0.999 | 0.999 | 1.000 | 0.999 | 0.999 | 0.990 | 0.991 |
| 9  | 0.999 | 1.000 | 0.995 | 0.987 | 0.997 | 0.999 | 0.999 | 0.999 | 1.000 | 1.000 | 0.985 | 0.985 |
| 10 | 1.000 | 1.000 | 0.995 | 0.986 | 0.997 | 0.999 | 0.999 | 0.999 | 1.000 | 1.000 | 0.987 | 0.987 |
| 11 | 0.987 | 0.988 | 0.993 | 0.996 | 0.994 | 0.991 | 0.987 | 0.990 | 0.985 | 0.987 | 1.000 | 0.995 |
| 12 | 0.988 | 0.987 | 0.993 | 0.989 | 0.995 | 0.991 | 0.986 | 0.991 | 0.985 | 0.987 | 0.995 | 1.000 |

**D**

|    | 1     | 2     | 3     | 4     | 5     | 6     | 7     | 8     | 9     | 10    | 11    | 12    |
|----|-------|-------|-------|-------|-------|-------|-------|-------|-------|-------|-------|-------|
| 1  | 1.000 | 0.942 | 0.934 | 0.859 | 0.899 | 0.924 | 0.978 | 0.987 | 0.936 | 0.973 | 0.862 | 0.852 |
| 2  | 0.942 | 1.000 | 0.942 | 0.957 | 0.933 | 0.973 | 0.942 | 0.967 | 0.986 | 0.969 | 0.888 | 0.833 |
| 3  | 0.934 | 0.942 | 1.000 | 0.911 | 0.946 | 0.951 | 0.903 | 0.961 | 0.908 | 0.930 | 0.900 | 0.883 |
| 4  | 0.859 | 0.957 | 0.911 | 1.000 | 0.936 | 0.952 | 0.850 | 0.904 | 0.909 | 0.887 | 0.945 | 0.835 |
| 5  | 0.899 | 0.933 | 0.946 | 0.936 | 1.000 | 0.973 | 0.844 | 0.945 | 0.873 | 0.904 | 0.960 | 0.957 |
| 6  | 0.924 | 0.973 | 0.951 | 0.952 | 0.973 | 1.000 | 0.896 | 0.963 | 0.936 | 0.954 | 0.937 | 0.884 |
| 7  | 0.978 | 0.942 | 0.903 | 0.850 | 0.844 | 0.896 | 1.000 | 0.966 | 0.958 | 0.976 | 0.809 | 0.767 |
| 8  | 0.987 | 0.967 | 0.961 | 0.904 | 0.945 | 0.963 | 0.966 | 1.000 | 0.949 | 0.983 | 0.901 | 0.887 |
| 9  | 0.936 | 0.986 | 0.908 | 0.909 | 0.873 | 0.936 | 0.958 | 0.949 | 1.000 | 0.969 | 0.812 | 0.765 |
| 10 | 0.973 | 0.969 | 0.930 | 0.887 | 0.904 | 0.954 | 0.976 | 0.983 | 0.969 | 1.000 | 0.851 | 0.810 |
| 11 | 0.862 | 0.888 | 0.900 | 0.945 | 0.960 | 0.937 | 0.809 | 0.901 | 0.812 | 0.851 | 1.000 | 0.918 |
| 12 | 0.852 | 0.833 | 0.883 | 0.835 | 0.957 | 0.884 | 0.767 | 0.887 | 0.765 | 0.810 | 0.918 | 1.000 |

**E**

|    | 1     | 2     | 3     | 4     | 5     | 6     | 7     | 8     | 9     | 10    | 11    | 12    |
|----|-------|-------|-------|-------|-------|-------|-------|-------|-------|-------|-------|-------|
| 1  | 1.000 | 0.941 | 0.933 | 0.858 | 0.899 | 0.923 | 0.978 | 0.987 | 0.934 | 0.972 | 0.862 | 0.852 |
| 2  | 0.941 | 1.000 | 0.941 | 0.957 | 0.932 | 0.973 | 0.942 | 0.967 | 0.986 | 0.969 | 0.887 | 0.832 |
| 3  | 0.933 | 0.941 | 1.000 | 0.910 | 0.946 | 0.951 | 0.900 | 0.960 | 0.906 | 0.929 | 0.900 | 0.882 |
| 4  | 0.858 | 0.957 | 0.910 | 1.000 | 0.935 | 0.951 | 0.849 | 0.903 | 0.909 | 0.887 | 0.944 | 0.833 |
| 5  | 0.899 | 0.932 | 0.946 | 0.935 | 1.000 | 0.973 | 0.843 | 0.945 | 0.872 | 0.903 | 0.959 | 0.957 |
| 6  | 0.923 | 0.973 | 0.951 | 0.951 | 0.973 | 1.000 | 0.895 | 0.963 | 0.935 | 0.954 | 0.936 | 0.883 |
| 7  | 0.978 | 0.942 | 0.900 | 0.849 | 0.843 | 0.895 | 1.000 | 0.966 | 0.957 | 0.976 | 0.809 | 0.765 |
| 8  | 0.987 | 0.967 | 0.960 | 0.903 | 0.945 | 0.963 | 0.966 | 1.000 | 0.948 | 0.983 | 0.902 | 0.887 |
| 9  | 0.934 | 0.986 | 0.906 | 0.909 | 0.872 | 0.935 | 0.957 | 0.948 | 1.000 | 0.969 | 0.811 | 0.762 |
| 10 | 0.972 | 0.969 | 0.929 | 0.887 | 0.903 | 0.954 | 0.976 | 0.983 | 0.969 | 1.000 | 0.851 | 0.808 |
| 11 | 0.862 | 0.887 | 0.900 | 0.944 | 0.959 | 0.936 | 0.809 | 0.902 | 0.811 | 0.851 | 1.000 | 0.917 |
| 12 | 0.852 | 0.832 | 0.882 | 0.833 | 0.957 | 0.883 | 0.765 | 0.887 | 0.762 | 0.808 | 0.917 | 1.000 |

Supplement: Additional file 5: — Technical replicate. Twelve replicate transcript counting libraries were prepared from 1 μg of a pool of wild-type zebrafish embryo total RNA sample. The libraries were sequenced by Illumina MiSeq and analysed using the DeTCT pipeline. The normalised counts for each region were extracted (73,938 regions). A. The coefficient of variance was calculated for all regions and plotted against the mean of the counts (blue, red and green circles) and the Pearson correlations shown in part B. Regions with a low mean count (i.e. with little or no chance of showing significant differential expression) were removed using DeSeq2 independent filter and the remaining 7,379 regions plotted on the same graph (red and green circles) and the Pearson correlations shown in part C. The proximity of the transcript counting 3′ ends (TC 3′ ends) was restricted to within 100 bases of an Ensembl transcript 3′ end and the resulting 1,976 regions plotted on the same graph (green circles) and the Pearson correlations shown in Fig. 3. The graph shows less dispersion between the filtered regions of the 12 technical replicates compared to the unfiltered regions. It was noted that one region mapping to the mitochondrial MT:2501-2518 (within ENSDARG00000080337) comprised a large proportion of counts, distorting the Pearson correlation (red arrow on A). We believe these are derived by priming from a polyA sequence in the mitochondrial rRNA sequence. The Pearson correlation of all regions with this outlier removed is shown in part D and after removing regions with low mean counts is shown in part E. Note this mitochondrial region was removed in the Ensembl transcript proximity filter and therefore does not appear in the Pearson correlation shown in Fig. 3. Cells coloured yellow in the Pearson correlation are the most highly correlated while those in blue are the least correlated. The yellow to blue gradient is specific to each individual Pearson correlation. (PDF 165 kb) [file 12864_2015_1788_MOESM5_ESM.pdf]

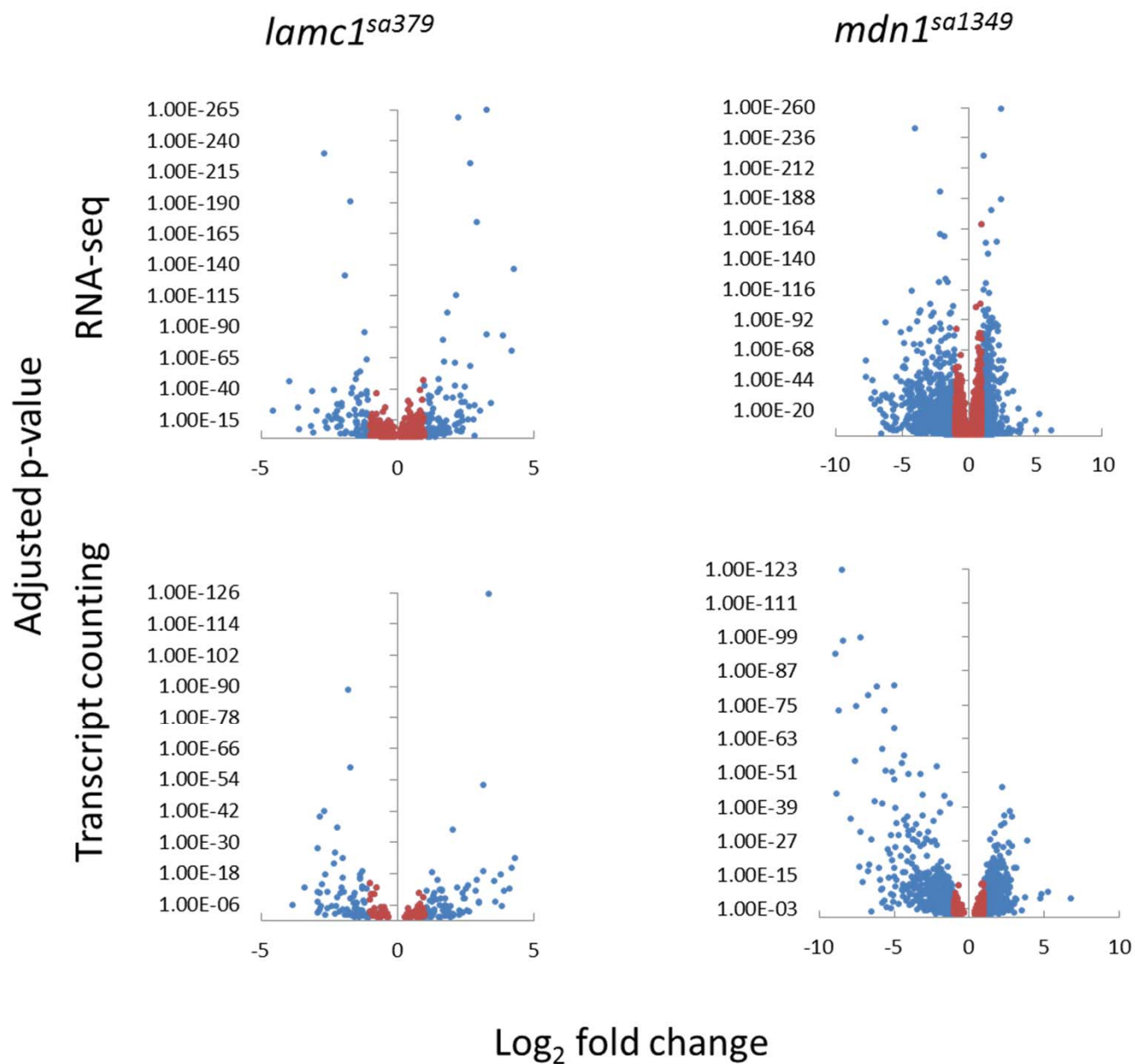

Supplement: Additional file 6: — Volcano plots of RNA-seq data. Volcano plots, plotting the adjusted p-value against the log2 fold change, are shown for the two knockout alleles analysed by RNA-seq and TC. All transcripts with an adjusted p-value <= 0.05 are shown. Transcripts with a fold change >= 2 are blue and a fold change < 2 are red. TC transcripts were passed through the relaxed proximity filter of -100 to +5000. (PDF 127 kb) [file 12864_2015_1788_MOESM6_ESM.pdf]
