# Supplementary material for: High-throughput and quantitative genome-wide messenger RNA sequencing for molecular phenotyping
Source: BMC Genomics. 2015 Aug 5;16(1):578. doi: 10.1186/s12864-015-1788-6 (PMC4524448; doi:10.1186/s12864-015-1788-6)
Supplement: Additional file 7: — Comparing RNA-seq and transcript counting. The same analysis as Fig. 5, but using mdn1 sa1349. (PDF 97 kb) [file 12864_2015_1788_MOESM7_ESM.pdf]

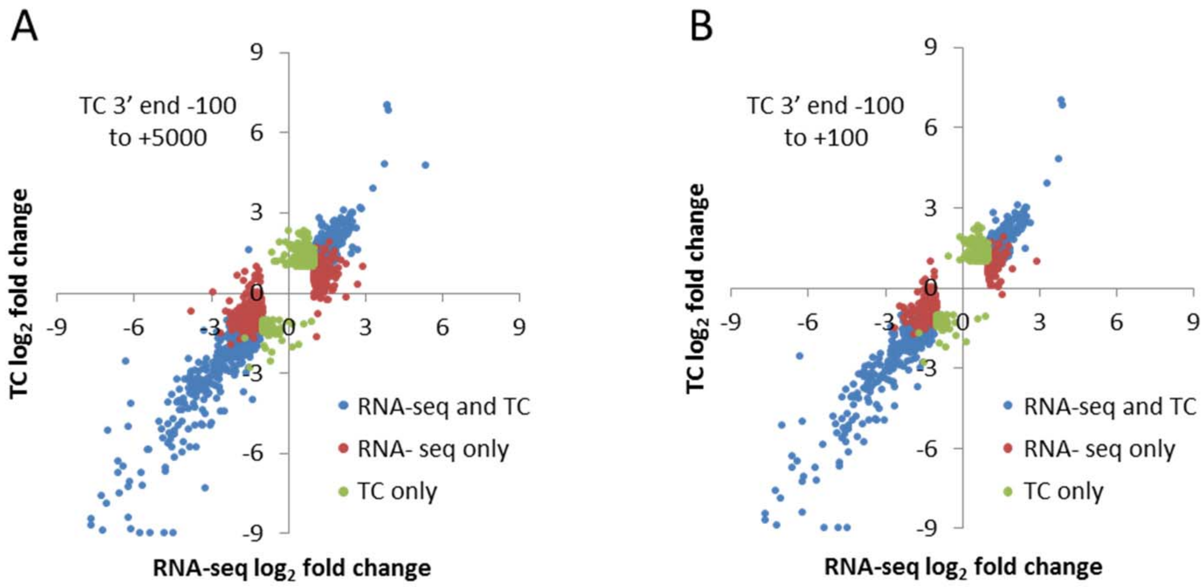

C

Number of genes in DE in *mdn1^{sa1349}*	TC 3' end -100 to +5000 from ENST	TC 3' end -100 to +100 from ENST
Both	961	742
RNA-seq only	482	289
TC only	466	363
Total	1982	1394
